# Clinical presentation of burning mouth syndrome in patients with oral lichenoid disease

**DOI:** 10.4317/medoral.23812

**Published:** 2020-08-27

**Authors:** Javier Alberdi-Navarro, José Manuel Aguirre-Urizar, Eduardo Ginestal-Gómez

**Affiliations:** 1 Oral Medicine and Oral and Maxillofacial Pathology Units. Dental Clinic Service, Department of Stomatology II, School of Medicine and Nursing, University of the Basque Country(UPV/EHU), Leioa, Bizkaia, Spain; 2 Orofacial Pain and Temporomandibular Disorders Unit. Dental Clinic Service, Department of Stomatology II, School of Medicine and Nursing, University of the Basque Country (UPV/EHU), Leioa, Bizkaia, Spain

## Abstract

**Background:**

To analyze the presence of burning mouth syndrome (BMS) in a group of patients diagnosed with oral lichenoid disease (OLD).

**Material and Methods:**

A retrospective study of 217 patients diagnosed with OLD; 158 (72,8%) women and 59 (27,2%) men, with an average age upon diagnosis of 56,4 years (SD 11,88). We carried out a detailed and complete characterization of symptoms, with special emphasis on BMS diagnostic data specified by the International Headache Society.

**Results:**

Four patients (1.8%) presented with long-term clinical symptoms of burning mouth, indicative of BMS and they fulfilled the IHS 2018 criteria, except for criterion D, i.e.“Oral mucosa is of normal appearance”. The observed lichenoid mucosal lesions were not considered to be able to account for the reported intraoral pain in any of our patients. Thus neither diagnosis was considered to be exclusive.

**Conclusions:**

Patients diagnosed with OLD, and who simultaneously present clinical characteristics of BMS should be studied in detail, in order to evaluate the possibility of both diagnoses concurring.

** Key words:**Burning mouth syndrome, oral lichen planus, oral lesions, oral lichenoid disease, burning pain.

## Introduction

Burning mouth syndrome (BMS) is a complex neuropathic disorder characterized by the presence of a chronic and constant burning-type pain which is generally bilateral, localized in the oral mucosa and frequently accompanied by other sensorial alterations, principally xerostomia and dysgeusia (1). Although the neuropathic nature of the condition was suspected right from the earliest descriptions of this syndrome (2) it is only in recent years that the neuropathic character of the syndrome has been clearly demonstrated, thanks to research and the application of a variety of neurophysiological techniques such as the blink reflex, quantitative sensory testing or functional magnetic resonance (3-5).

The concept of BMS has undergone numerous modifications over the years. Thus initially, an idiopathic primary BMS was identified, as well as a secondary BMS associated with local or systemic etiopathogenic factors (6,7). This approach made management of these patients rather complex since they required different complementary tests, some of which were rather complex, as well as a detailed analysis of all the possible factors which could condition the appearance of the painful oral symptomatology (6,8). Due to the neuropathic nature of the disorder and the lack of clinical evidence of the participation of local and/or systemic factors in the genesis of BMS, it was decided in the third classification of the International Headache Society (IHS) (1) that BMS be considered to be a primary process which did not require the ruling out of etiopathogenic factors for its diagnosis; this approach was, nevertheless, not shared by the International Association for the Study of Pain (IASP) (9). The IHS considers that the oral mucosa of the patient diagnosed with BMS should have a normal appearance, with this criterion remaining unchanged with respect to the previous classification (1,7).

Thus, the presence of oral lesions in BMS patients remains somewhat controversial.

Recently, a number of authors have reported patients with both oral lichenoid disease (OLD) and BMS (10).

OLD is an immunologically-based, chronic mucous condition which affects mainly peri-menopausal women. It is characterized by the appearance of white papule lesions, occasionally with a reticular, net-like pattern, which can be accompanied by other lesions (11,12). When patients with OLD present with “white” mucous lesions only, preferentially reticular papules, the condition usually evolves without pain, whereas when atrophic and above all erosive-ulcerated lesions appear, discomfort and pain are usually reported (13). Normally discomfort is mild and non-specific and often reported as a “sensation of roughness” which is exacerbated upon ingesting hot or acidic food (13). The pain associated with OLD is located in the superficial mucous and it has been reported to exhibit variable intensity depending on the affectation and on the functional stimulation of the lesioned mucosa, as well as on the ingesting of acidic, hot or spicy foods (14).

In the light of the clinical characteristics of these two disorders and their possible simultaneous appearance, we decided to analyze the presence of BMS in a group of patients diagnosed with OLD, identifying key characteristics of these patients.

## Material and Methods

Retrospective study of 217 patients clinicopathologically diagnosed with OLD at the Oral Medicine and Oral and Maxillofacial Pathology Units which pertain to the Service of Clinical Odontology of the University of the Basque Country (UPV/EHU), between the years 1999 and 2014. The sample is made up of 158 (72.8%) women and 59 (27.2%) men, with an average age of 56.4 years (SD: 11.88) at the time of diagnosis, with a minimum of 21 and a maximum of 90 years.

This study was approved by the Ethics and Research on Human Beings Committee of the University of the Basque Country (UPV/EHU; CEISH185/2012). In all cases, we carried out a clinicopathological characterization of OLD based on established criteria (11,12,15). We also carried out a precise evaluation of the oral symptoms, paying particular attention to those which could be related to BMS. Following the Dental Clinic Service protocol, patients with symptoms indicative of BMS were referred to the Orofacial Pain and Temporomandibular Disorders Unit and were studied using a specific protocol for diagnosis and characterization of the syndrome. In all patients, we ruled out the existence of other local and systemic pathologies by carrying out a complete oral and maxillofacial exploration, as well as salivary and mycological study and blood tests. A descriptive statistical analysis was performed with the data obtained.

## Results

In accordance with clinicopathological criteria previously established for OLD (12,13,16) 162 (74.7%) patients were classified as presenting with oral lichen planus (OLP) and 55 patients (25.4%) with oral lichenoid lesion (OLL). Table 1 shows the principal demographic data of the study as well as some clinical characteristics. In this OLD sample, more than half of patients (50.7%) presented with discomfort and/or pain upon being diagnosed. The majority of these patients (65.5%) reported non-specific discomfort and the remaining 34.5% complained of differing degrees of mucosal pain. Finally, 4 patients (1.8 %) who had been diagnosed with OLD presented with daily oral pain which was burning in character, had lasted for more than 6 months and constituted 10.5% of pain cases. These patients fulfilled the diagnostic criteria for BMS (1), with the exception of part of criterion D which indicates that “oral mucosa is of normal appearance”. The principal clinical and symptomatic data of these OLD-BMS patients are presented in Table 2. These cases were called to clinic and an examination was performed in January 2020, in which the data of pain symptoms and the clinical location of the lesions were corroborated, verifying that they had not been modified. Furthermore, the clinical course of pain was continued and independent of the evolution of the OLD lesions.

**Table 1:**
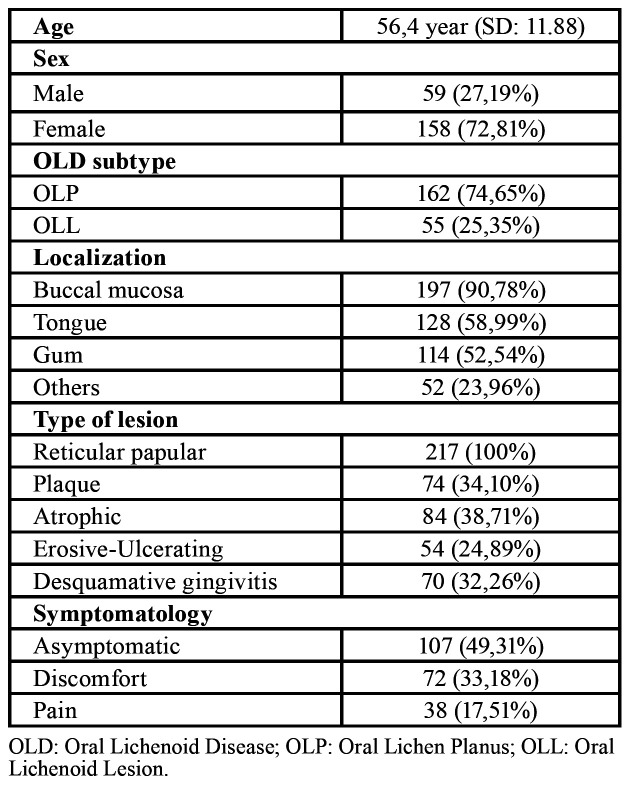
Clinical data of the study sample.

**Table 2:**
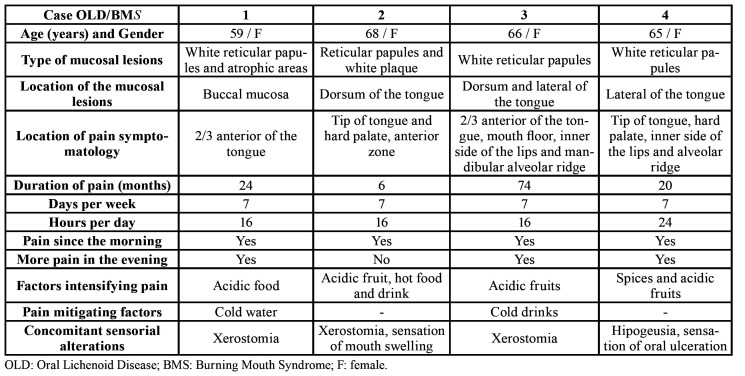
Symptomatic characterization of patients with OLD and BMS.

## Discussion

In 2004, the first diagnostic criteria for BMS were established on behalf of the IHS (7). These involved a diagnosis of exclusion in which it was necessary to rule out possible systemic and local processes which could condition the appearance of intraoral burning, but without establishing a time of minimum duration. In addition, the oral mucosa should have a normal appearance and all recognizable local and/or systemic causes should be ruled out. In the recent 2018 classification of the IHS (1), a temporal parameter for pain has been established, i.e. for more than 2 hours per day and for more than 3 months, which has helped rule out other short-term pathologies. However, this new classification continues to insist that the oral mucosa be of normal appearance.

The IASP (9) continues to indicate that local and systemic causes must be ruled out in order to be able to establish a diagnosis of BMS, a principle which is shared by the majority of research studies carried out to date (16-18). Among the reported local causes are the oral diseases which can give rise to intraoral pain, such as OLD. In this regard, the IHS (1) and the majority of clinical studies of BMS insist that the “oral mucosa is of normal appearance and clinical examination including sensory testing is normal” thereby excluding all those patients who present with diseases of the oral mucosa, as is the case of patients with OLD.

In recent years, a number of patients diagnosed with oral lichen planus have been reported to present with reticular white papular lesions in the oral mucosa and with symptoms similar to those of BMS (10). These patients, similar to those reported in the present study, would not receive the diagnosis of BMS if we applied the current diagnostic criteria proposed by the IHS (1).

BMS and OLD share a series of demographic characteristics, such as the age of appearance of both processes, i.e. between the 5th and 7th decades of life, as well as their gender distribution, with both pathologies being more frequent in women (19-23). In addition, the prevalence of these oral disorders is also similar; OLD affects 1.27% of the population (24), whereas BMS affects between 1 and 3.7% (22).

Overall, when a wide range of samples of both processes are studied, as in the present study, the resulting data are indicative of a certain degree of coincidence. Thus, almost 2% of patients clinicopathologically diagnosed with OLD can also have BMS. Consequently, we believe that none of the diagnoses should be ruled out in these cases, and patients should be treated for both disorders, as has been similarly reported for other concurring processes such as BMS with the Sjögren syndrome or with other systemic pathologies (25,26).

In addition, the prevalence of BMS found in our sample of OLD is within the range reported for the general population (22). Nevertheless, we have the impression that this comorbidity may in fact be higher, simply due to the fact that both processes principally affect post menopausic women.

It is important to attempt to distinguish both diseases, taking into account the characteristics of the painful symptomatology presented by patients with OLD, including the quality of the pain, its localization, duration and frequency, as well as modifying or conditioning factors. We should also take into account the topographic relation existing between the OLD lesions and the symptoms which are present, as well as the types of mucosal lesion which these patients exhibit. In our study, none of the patients with OLD and BMS exhibited a direct relation between the localization of the lichenoid mucosal lesions and the pain which they reported. Moreover, regarding the type of mucosal lesions present in these patients, the majority were reticular papules or white plaques, rather than atrophic, erosive or ulcerated lesions, as would have been expected and has been reported in symptomatic cases of OLD (27). These data are indicative of the simultaneous presence of both disorders in the same patient. In addition, the prevalence of BMS found in our sample of OLD is in the range described for the general population (22) and therefore it was statistically predicTable.

Another important differential characteristic of these patients with both OLD and BMS is the duration of the symptomatology, as well as the presence of other symptoms in addition to pain. Concerning symptom duration, in the four cases reported here, pain was continuous and had lasted for more than 6 months. This is more a characteristic of BMS pain rather than a characteristic of oral mucosal pain (22). Regarding other symptoms, in all cases we recognized the presence of accompanying sensorial alterations, with xerostomia being the most frequent, in keeping with the typical clinical characteristics of BMS (22).

To conclude, the results of our study show the existence of patients with both OLD and BMS. Consequently, we consider that it is not appropriate to exclude in a systematic way the diagnosis of BMS in those patients who present lesions in the oral mucosa. On the basis of these results, we think that the redaction of the criterion D of the IHS “oral mucosa is of normal appearance and clinical examination including sensory testing is normal” is adequate for clinical investigations protocols. However, this criterion could be too restrictive for correct BMS diagnosis in daily clinical practice. On the other hand, the description of the clinical presentation of BMS by both the IHS and the IASP (1,9), in which it is indicated that there are no lesions that justify burning pain would allow the diagnosis of both processes. This modification of the clinical evaluation of BMS patients would facilitate a more adequate treatment of these cases.

We are aware of the limitations of our study, although it is true that it is difficult to design a prospective study given the low chance of finding both diseases in the same patient.

Finally, we would like to insist on the necessity of always carrying out a complete exploration of the oral mucosa in these patients in order to evaluate the existence of mucosal lesions, before ruling out a BMS diagnosis, as can occur in some patients diagnosed with OLD. It should not be forgotten that OLD is a potentially malignant disorder of the oral mucosa and that all of the lesions present in these patients should be analyzed, treated and controlled in an appropriate way, always paying attention to the symptomatology reported by the patient.
